# The use of evidence in English local public health decision-making: a systematic scoping review

**DOI:** 10.1186/s13012-017-0577-9

**Published:** 2017-04-20

**Authors:** Dylan Kneale, Antonio Rojas-García, Rosalind Raine, James Thomas

**Affiliations:** 1 Evidence for Policy and Practice Information and Coordinating Centre, UCL Institute of Education, University College London, 20 Bedford Way, London, WC1H 0AL UK; 2 NIHR CLAHRC North Thames, Department of Applied Health Research, University College London, 1-19 Torrington Place, London, WC1E 7HB UK

## Abstract

**Background:**

Public health decision-making structures in England have transformed since the implementation of reforms in 2013, with responsibility for public health services and planning having shifted from the “health” boundary to local authority (LA; local government) control. This transformation may have interrupted flows of research evidence use in decision-making and introduced a new political element to public health decision-making. For generators of research evidence, understanding and responding to this new landscape and decision-makers’ evidence needs is essential.

**Methods:**

We conducted a systematic scoping review of the literature, drawing upon four databases and undertaking manual searching and citation tracking. Included studies were English-based, published in 2010 onwards, and were focused on public health decision-making, including the utilisation or underutilisation of research evidence use, in local (regional or sub-regional) areas. All studies presented empirical findings collected through primary research methods or through the reanalysis of existing primary data.

**Results:**

From a total of 903 records, 23 papers from 21 studies were deemed to be eligible and were included for further data extraction. Three clear trends in evidence use were identified: (i) the primacy of local evidence, (ii) the important role of local experts in providing evidence and knowledge, and (iii) the high value placed on local evaluation evidence despite the varying methodological rigour. Barriers to the use of research evidence included issues around access and availability of applicable research evidence, and indications that the use of evidence could be perceived as a bureaucratic process. Two new factors resulting from reforms to public health structures were identified that potentially changed existing patterns of research evidence use and decision-making requirements: (i) greater emphasis among public health practitioners on the perceived uniqueness of LA areas and structures following devolution of public health into LAs and (ii) challenges introduced in responding to higher levels of local political accountability.

**Conclusions:**

There is a need to better understand and respond to the evidence needs of decision-makers working in public health and to work more collaboratively in developing solutions to the underutilisation of research evidence in decision-making.

**Electronic supplementary material:**

The online version of this article (doi:10.1186/s13012-017-0577-9) contains supplementary material, which is available to authorized users.

## Introduction

Since 2013, the context in which local public health strategy is developed and services are commissioned in England has shifted, and decisions previously made within National Health Service (NHS) structures are now being taken by different organisations and stakeholders. The shifting culture and context of decision-making means that as generators and synthesisers of research evidence we need to respond to these changes if we are to continue to support public health decision-makers to make informed and judicious evidence-based choices [1].

**Fig. 1:**
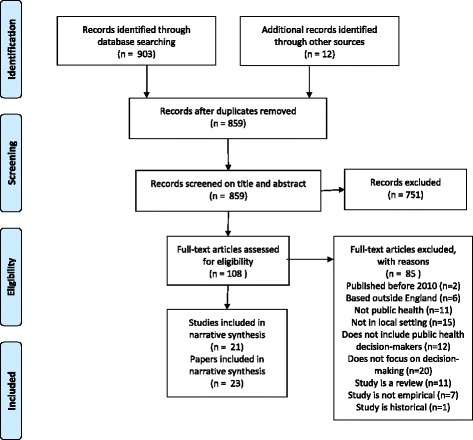
Flow of studies through the review

The improvement of public health outcomes through evidence-based strategies is widely recommended, offering greater access to information on what works, increased opportunities for the effective use of resources, and improved certainty around the likelihood of success implementing different intervention options [2]. There is no shortage of examples where evidence assembled from different settings has been used to directly inform and mobilise public health interventions, the implementation of which has led to substantial improvements in health behaviours and health states on a large scale. For example, Ireland’s trailblazing workplace smoking ban [3] which led to attributable increases in smoking cessation [4] was developed on the basis of a strong evidence base [5]. Conversely, the potential for ineffective or detrimental public health interventions to pose harm on a large scale provides further justification of the importance of effective use of evidence (see [6, 7]). Indeed, some authors highlight that the use of “scientific knowledge” is an integral dimension of the very definition of public health, which involves the “process of mobilizing and engaging local, state, national, and international resources to assure the conditions in which people can be healthy” ([8], p538).

In the case of public health, the complexity in targeting populations, or communities, necessitates providing evidence that is both comprehensive and sensitive to this challenge [9]. It follows that the complexity of this evidence may lead to challenges in its effective implementation [10, 11].The shift in decision-making structures means that not only do we need to understand the new culture and practices of evidence use in decision-making but we also need to examine critically whether our own research outputs are fit for purpose. The systematic scoping review presented here explores how research evidence is being used in public health.

### How has the structure of local public health decision-making changed?

Since 2013, local public health leadership has mainly transferred to local authorities (LAs), whose public health remit now includes commissioning services across most aspects of public health (see [12] for a full outline). LAs are also responsible for improving health determinants and reducing health inequalities across nine key areas impacting on population health including early years, education, planning, housing, leisure and communities [13]. This was said to be emblematic of a “social model of health” [14], with LAs having an interest, and potentially an ability to influence, most local public health activities and many local determinants of health. Newly created health and wellbeing boards provide some strategic leadership, support, and coordination on the response of LAs to local health challenges. Most other local health services are commissioned through local clinical commissioning groups (CCGs; which replaced primary care trusts (PCTs); although some aspects of public health are retained here).

Although Table 1 attempts to disentangle what is a fiendishly complex public health delivery landscape, actual day-to-day practice means that the divisions in responsibilities displayed below may not necessarily be as clear-cut in real-world settings. For instance, screening and immunisation programmes are commissioned by the NHS England and its regional teams. Directors of Public Health and LA public health teams have a responsibility to provide advice and information to inform immunisation and screening plans, as well as to scrutinise these [15]. Implicitly, this means that while the commissioning of immunisation and screening services is ostensibly an NHS endeavour, in practice, the delivery of immunisation and screening services is contingent on both LA and NHS input and resources. Furthermore, the LA remit could extend much further in some cases (for example to include quality assurance) when the delivery of such services is in turn reliant on agencies that lie within other LA commissioning areas. Therefore, while Table 1 provides an indication of the broad areas of responsibility across different agencies, the descriptions provided cannot represent the full extent of dependencies between agencies.

**Table 1:** Main agencies involved in public health post-2013

Name	Geographic remit (in England)	Post-2013 broad responsibilities^a^
Organisations with statutory duties		
Local authorities (LAs)	Local	Responsible for planning and commissioning most local public health services (see above)
Clinical commissioning groups (CCGs)	Local	Retain some related public health functions including provision of Child and Adolescent Mental Health Services (CAMHS) and mental health services, occupational health, maternity services and commissioning of alcohol workers in various settings (although overall responsibility for alcohol misuse services, prevention and treatment sits with LAs) [64]. (221 CCGs)
Health and wellbeing boards (HWBs)	Local	Coordinate activity of local health and care leaders to improve population health, reduce health inequalities and introduce democratic accountability [65].
National Institute for Health and Care Excellence (NICE)	National	Issues guidance on the effectiveness of interventions that can improve population health and reduce health inequalities.
Public Health England (PHE)	National	Provide support, epidemiological guidance and research for LAs and coordination of national public health initiatives and campaigns; includes former Public Health Observatories
Greater London Authority (GLA); Greater Manchester Public Health Network (GMPH) (and other regional organisations)	Regional	GLA issues guidance and collects and synthesises epidemiological and demographic research for London Boroughs and also issues various toolkits for action.
		GMPH is a collaborative network of ten directors of public health working together to achieve goals that could not be realised individually.
		Other regional organisations and networks exist.
NHS England/NHS Commissioning Board	National	Provides oversight of CCGs; specific functions around the commissioning of primary care; retained some screening functions.
Department of Health	National	Retains stewardship over relevant agencies including Public Health England, NHS England and the Health and Social Care Information Centre.
Other types of organisation		
King’s Fund, Nuffield, Local Government Association, and others	National	These organisations produce influential information and guidance directly for local public health decision-makers; they are an important component of the new evidence and decision-making landscape

^a^See explanatory text above for caveats around dependencies in commissioning services and potential co-commissioning

The transition to new health decision-making structures described above is marked by heterogeneity (e.g. [16]), which is in part reflective of existing differences in local authority needs and practices [17]. The impact of this heterogeneity on public health decision-making practices and actions is difficult to quantify; although, there have been some tangible actions common to all areas. First, all local authorities were expected to appoint a Director of Public Health (DPH) responsible for the public health budget and creating a staff of public health consultants. Second, local authorities were jointly tasked with producing/ updating Joint Strategic Needs Assessments (JSNAs), whose purpose is to describe the current and future health needs of the population. A third tangible action was the creation of Health and Wellbeing Strategies (HWSs), produced by HWBs, which present a response outlining strategies and services that would be commissioned to address the needs identified within JSNAs [18].

### Implications for evidence use

The transition to a new landscape of decision-making is perceived to have progressed reasonably smoothly in some quarters [19], even if future challenges have been identified including anticipated further squeezing of public health budgets. However, this standpoint is not universal across professionals and academics working in public health, and more recent evidence highlights substantial levels of concern about ongoing risks and severe challenges to the local delivery of public health [20].

Structural changes have implications for the way in which research evidence is used in public health decision-making. For example, the appointment of DPH exposed differing levels of commitment between those LAs who followed DH guidance and created new senior directorship posts for DPH and those with different interpretations [21]. Furthermore, reflective of the nature of LAs, final decision-making responsibility ultimately now falls with the elected members, not with officers [22, 23]. Consequently, public health is now exposed to a culture that is more likely to be shaped by political and legal constraints [24] and from an evidence-use perspective, an increased political element to local decision-making may have implications in terms of the type and format of evidence needed. A political dimension can also make public health priorities vulnerable to rapid changes due to transitions in power, and producers of evidence will need to respond quickly to support new directions or provide evidence to justify existing activities. Reductions in public health grants supporting the extensive public health remit of LAs [14] and the different interpretations around how ring-fenced public health monies should be spent [25] are also likely to influence the evidence required.

Within the extensive literature on evidence-informed public health policy-making more broadly, a pattern of underutilisation of research evidence persists despite its abundance [26, 27] and despite the recurring cycles of prioritisation by policy-makers [28]. Common barriers to evidence use include perceived limitations in the relevance, importance and credibility of evidence [24, 29], including concerns around the application of evidence generated in other settings [24, 30]. A previous review concluded that few studies actually reveal the process through which research evidence is used in public health decision-making [31], and how this sits within broader knowledge-utilisation frameworks. In addition, the use of the word “evidence” is not necessarily synonymous with drawing upon a robust body of research evidence [32]. Where researchers have started to examine these issues in the post-2013 English landscape, they have indicated continued difficulties in using research evidence. In Beenstock and colleagues’ [18] analysis of 47 HWSs, they found that very few (5) referred to research evidence published in academic journals, just three referenced NICE guidance, and none directly referenced a Cochrane systematic review. Furthermore, research evidence was used more commonly to demonstrate evidence of need than to demonstrate evidence of effectiveness.

The aim of the review presented here is to offer further insight on these issues through mapping the use of research evidence in public health decision-making at a sub-national level, and where possible to compare patterns of evidence utilisation before the reconfiguration of public health services (2010–2012/13) and afterwards (2013/14–2016). Unlike previous reviews, this review adopts a broad set of inclusion criteria around study design, with both observational and experimental studies included, but a narrow scope in terms of geography and date range. Nevertheless, the patterns of evidence use and the obstacles that decision-makers in local areas face when sourcing and using evidence are expected to have a much broader resonance and be of interest to readers outside England. This study was conducted by a team of systematic reviewers who are interested in the contribution of systematic reviews to public health decision-making in England, and how this evidence contributes alongside other sources of research evidence and public health intelligence more broadly.

## Methods

### Search strategy

Four databases were searched for studies published between 2010 and 2016 (June) (PubMed, HMIC, EconLit and Scopus) and specific search strategies were designed for each (see Additional file 1). In addition, a manual search of databases was carried out in order to find potentially relevant studies. A bibliographic database was created in EPPI-Reviewer 4 to store and manage the references [33], and data were extracted into Microsoft Excel.

### Assessment of eligibility

Titles and abstracts of the documents retrieved in the searches were independently screened by two reviewers to determine eligibility (full inclusion criteria provided in the Additional file 2). Included studies were England-based studies published from 2010 onwards that were focused on public health decision-making in local (regional or sub-regional) areas. The search strategy was limited to studies that included “public health” (or “health promotion”) in their title, abstract or keywords. Public health decision-making was defined broadly as that which aims to promote and protect the health and wellbeing of groups, communities and populations, mirroring broad definitions employed in previous reviews [31]. We did not extend this definition to explore decision-making around the social determinants of health (e.g. education or employment inequalities) except when this was described as taking place specifically within the context of public health (see [34] for an example where this took place). Included studies focussed on decision-making involving (i) local public health services or (ii) local public health prioritisation (using LA’s commissioning responsibilities [12] as a guide where there was any ambiguity), or (iii) explored local decision-making among professionals working in public health. Studies should have directly included decision-makers and a focus on the process of evidence use in decision-making. Studies published during 2010 and onwards were selected for inclusion. This date range was selected to provide evidence of research use practice immediately before and after the enactment of reform to decision-making structures in England (and where possible to contrast these); 2010 also coincides with the publication of the first systematic review by the Cochrane Public Health group, a recognition of the need for greater applicable evidence on specialist complex community-based interventions to contribute to the evidence-based policy movement. Where other systematic reviews were encountered, these were examined primarily as a further source of studies. We also excluded studies that did not directly involve observations of decision-makers (including [18]). Studies fulfilling the inclusion criteria were selected for full-text assessment, after which a new independent assessment was performed. Disagreements were resolved through discussion between the reviewers.

### Data extraction and synthesis of the results

After piloting, we extracted information from all included studies. We did not undertake formal quality assessment of the studies since the aim of the review is to map the literature in this area and to lay the groundwork for primary research and more detailed synthesis (if supported by the data). This means that we did not formally employ existing taxonomies of implementation to structure or guide our synthesis; although, our aims align with an ambition of understanding the processes of translating research into practice (developing process models) and understanding influencers on evidence use (developing determinant frameworks) [35]. In this systematic scoping review, our synthesis methods were confined to a narrative, configurative approach [36] and we intended to provide a descriptive account of the main recurring themes. We followed five stages: (a) initial coding the text by producing preliminary textual descriptions of studies and their findings in a tabular format (see Additional file 3); this also involved grouping the studies according to their characteristics (e.g. setting and stakeholders) in order to understand the characteristics of the body of literature and to observe emerging patterns in the data; (b) further inductive coding of the textual summaries and identifying key preliminary themes and their recurrence across studies; (c) developing a framework for arranging groupings and clusters of studies according to the themes and exploration of these within and between the studies; (d) further generation of analytical themes through attempting to develop a common rubric to describe these findings; (e) finally, although we did not formally quality assess the robustness of individual studies, we did consider the completeness and applicability of evidence, the robustness of the synthesis methods and the quality of evidence in terms of its relevance to the ambitions of the review, and this is presented in our discussion [37–39]. This process was carried out by two of the authors (DK and ARG), and any disagreement was also resolved by discussion.

## Results

The search retrieved 903 references, 43 of which were identified as duplicates. After examining the abstracts and titles of the retrieved studies, 108 potentially relevant papers were selected for full-text assessment. The papers for these 108 studies were retrieved and were subject to a second round of full-text screening from which we identified a total of 23 papers from 21 studies for synthesis (including two discovered through manual searching (see Fig. 1)). Their characteristics are summarised in Table 2, and their main features in relation to the use of evidence are summarised in the appendix tables, with themes emanating from these discussed below. Most studies were carried out in England (14); a surprisingly high number purported to cover the UK (7) despite public health policy being largely devolved. Few studies (8) were conducted during or after the implementation of the HSCA 2012 (in April 2013) while almost three-quarters of the studies were conducted with stakeholders based in different institutions (namely LAs, PCTs, CCGs).

**Table 2:** Characteristics of studies

	*N* = 21^a^	%
Region		
UK	7	33%
England	14	67%
HSCA 2012		
Before implementation (pre-April 2013)	12	57%
After implementation (post-April 2013)	6	29%
Before and after	2	10%
Unclear	1	5%
Setting		
Various (NHS, LA, PCT, etc.)	15	71%
Local authorities only	5	24%
PCTs only	1	5%
Type of evidence		
Specific programmes/interventions	1	5%
Economic evidence	2	10%
General research evidence	18	86%

^a^Refers to studies not papers; 23 papers included

### Locality of evidence

Many studies suggest that the research evidence base does not match the evidence needs of decision-makers with respect to the locality of evidence [34, 40–44]. This was identified as an underlying reason why decision-makers consult with sources other than research evidence [34, 40, 41]. In one of the included studies, having local evidence was said to “trump” other forms of evidence, even if this is of lower methodological quality [41]. For example, Wye and colleagues [41] contrast the low impact that a briefing based on academic research evidence had among a committee considering commissioning telehealth, when compared to experiential local evidence based on eight service users. Despite the methodological robustness of the former, the latter evidence refuted the findings of the former and was instrumental in increased positivity towards telehealth among the committee [41]. A similar theme was shared among other studies where contextually relevant evidence was prioritised in decision-making [34, 45], including local public health intelligence [34, 42, 46]. Problematically, decision-makers may emphasise the uniqueness of their local areas and their public health challenges, as opposed to highlighting commonalities with other areas [40].

In some studies, “local” denotes geographically bounded evidence that would allow for service planning (evidence of need) or benchmarking or drawing comparisons of effectiveness with neighbouring areas (based on evidence of effectiveness) [34]; in other studies, “local” appeared not to be strictly geographically bounded but involved a broader consideration of evidence that was contextually salient [42].

### Decision-making and local contexts and local conceptualisations of public health

Evidence that was aligned to the local political ideology [40, 47] and could support broader organisational aims (where benefits could be visible beyond public health alone [43, 48]) was most useful to decision-makers. Philips and Green’s study, for example, which aims to understand the processes and practices of local public health decision-making, highlights how decision-makers construct unique identities for their organisations that translated into specific processes for action, which in turn led to perceived distinctiveness in terms of information needs [40]. One study found that services and strategies did not always need to be supported by evidence if these initiatives were congruent with the current direction of the organisation [49]. This political ideology was shaped by the strong affiliation with accountability to local populations, and sometimes, this could lead to conflicting perspectives as to which public health issues to prioritise. Marks and colleagues [14] provide a compelling example from a HWB board member highlighting the tension between evidence and local accountability: “…it was pointed out that lots more people die of smoking related conditions than they do of drug related conditions, alcohol and drug related conditions, but nobody complains to me about the next door neighbour smoking. But they will complain about the drug dealers on the corner and the alcohol, noise and abuse and all that stuff, which has a big effect on peoples’ lives. It ripples out on the community. But they’ve got a point, but we’ve got a point as well. (p1200)” Political considerations and public perceptions directly shaped elected members’ decision-making, even where there existed robust evidence to suggest an alternative course of action, as articulated in one study where a DPH found challenges in “advocating something [in this case minimum alcohol pricing] at a population level that is just not palatable from a political” ([44], p32).

One study, which took place after 2013, also found that the local political ideology and the type of case that was required to made around a given public health issue changed over a short duration [49], which may impact on the type of evidence required to support, or in some cases challenge this.

### The value of expert opinion

The need for evidence that was transferable to the local political context may also be an underlying explanation as to why expert opinion, advice, and experience were found to be highly utilised and valued in several studies [42, 46, 50, 51]. Expert opinion and advice was sometimes utilised more than other robust forms of evidence, including NICE and other national guidelines [41, 46] and systematic reviews and meta-analyses [42]. Experts often included professional colleagues, suggesting that expertise may be again related to experiential knowledge [46].

There were some indications of conflicting evidence-use patterns, with usage patterns not necessarily reflective of importance. One study found that NICE guidelines were one of the most frequently used sources of evidence, but practice guidelines (including NICE guidelines) were not universally regarded as the most useful [42]. The sample composition and the range of roles and specialisms may have partially accounted for some of this discrepancy between perceptions of usefulness and frequency of usage; although, the study did not publish disaggregated results that allow this to be interrogated further. This study also suggested that expert opinion was one of the most frequently utilised sources of evidence, but one that was not always highly valued [42].

Social network analyses suggest that the most powerful influencers on public health decision-making were able to form a bridge between local authority and NHS organisations [51]. In contrast, academic bodies and individual academic experts were rarely considered to be influential in their own right [51], perhaps reinforcing the findings of other studies which emphasise the disconnect between academics and policy-makers with regards to what constitutes useful and robust knowledge [52] as well as the broader disconnect between cultures of public health evidence generation and evidence use [53, 54].

### Evaluation evidence, experts and localism

A number of studies suggested that some of the most influential evidence on local public health decision-making was generated through local evaluation activities [40, 41, 55]. Such data meet decision-makers’ requirements around the locality of evidence and are generated within the local political context therefore meeting requirements around the transferability of findings [40, 47]. One study reported that evaluation evidence, even if based largely on an anecdote or a very small number of cases, could sway senior decision-makers’ views [41]. Despite the local evaluation evidence being generally regarded as useful for decision-makers, some studies also identified that this form of evidence had limitations particularly around the timing and intended usage [45, 56].

The degree to which the demand for and usage of evaluation evidence, evidence from experts, and locally embedded evidence are linked trends is not clearly expressed in the literature. Frequent deployment of local experts in public health decision-making may be as a result of their ability to blend inter/national sources research evidence with knowledge gained from local evaluation/experience. Oliver and DeVocht [42] identified a potentially broad knowledge translation role for reliable experts in public health decision-making in explaining the importance of findings, translating evidence into contextually “comprehensible statements” and “providing clear direction for decision-makers”. This suggests that the remit of experts in this case could extend beyond the usual boundaries of knowledge translation [57] to a much more directive role. The data they collected in the study did not illuminate the characteristics or motivations of existing experts or precisely the type of evidence where a knowledge translation role was most desired. However, they did note that decision-makers expressed a desire to utilise existing data and evidence through improved interpretation, suggesting that further refinement of knowledge translation practice was desirable [42].

### Demand for economic evidence falls short of the mark

Several studies suggested that decision-makers would find evidence around the economic impacts of interventions useful, but that this was not always available [14, 45, 47, 49, 53, 55]. In some cases, an increased demand for evidence around cost and benefit implications was directly related to austerity and the prioritisation that local decision-makers would now have to undertake as a result of reduced, and in some cases, unprotected budget [14]. However, not all methods of prioritisation based on economic methods are viewed as being sensitive to local contexts and some are viewed as excessively technocratic [14].

Two studies provided further disaggregation around the type of economic evidence that decision-makers valued the most [48, 58], which included evidence of impact across the remits of local authorities beyond traditional departmental siloes [48] as well as evidence of the way in which existing local services and structures were likely to influence cost effectiveness [58]. Marks and colleagues [14] provide an outline of different potential approaches and tools that can support decision-making and prioritisation and map these onto different stages of decision-making. For example, the first decision-making stage involves reaching an agreement on public health objectives; here, relevant information includes JSNAs and other public health intelligence, while other decision-support methods that may be employed include broad stakeholder consultation and involvement. Further stages of decision-making outlined include identifying options and resources for reaching the objectives, identifying measurable criteria, deciding on preferences and making choice. This study provides one of the few examples where authors have tried to understand decision-making as a series of sequential processes. However, they do not present explicit information on levels of awareness or usage of the different decision-support tools, or how much resonance the stages of decision-making hold across the three localities included in the study.

### Solutions, facilitators and barriers identified in the literature

One study identified evidence briefings based on systematic reviews as a promising but currently underutilised approach [40] while a further study suggested methodologically robust case studies of local innovation as being a way of enhancing the usefulness of academic research evidence for policy-makers [34]. Other studies identified strengthening networks and communications between evidence producers and evidence users, including recognising the role of interpersonal relationships in determining evidence use and influence, as being important in working towards meeting the evidence needs of public health decision-makers [42, 48, 51, 52]. None of the studies specifically mentioned “co-production” as a term; although, some recommended greater collaboration between generators of evidence and evidence users [52, 56]. For example, one study suggested that different “cultures of evidence” were not incommensurable in collaborative work between evidence producers and users when the gains to be made from collaborative working were made clear to all parties involved [56].

Named barriers to evidence use included access [41], capacity to analyse and interpret evidence [42], availability and relevance [58] and knowledge of different sources and types [42]. In terms of economic evaluations, another study also highlighted the difficulties to analyse the return on investment due to the organisational culture, capacity, the status of services, or administrative or political inertia [48]. A final study provided indicative evidence that the use of evidence was perceived as being tied to other decision-making processes (such as strategic partnership working) that were perceived as “bureaucratic” [59].

## Discussion

### Summary

This systematic scoping review identified 21 studies (23 papers) that included a specific focus on the process of English public health decision-making from 2010 onwards. These studies allowed us to identify some recurring themes around the role research evidence plays in decision-making, as well as some of the major influencers on the use of research evidence use. As we discuss below, few studies provided descriptive accounts of the way in which research evidence is used in practice, which precluded the development of process models representing the way in which research evidence is translated into local public health practice [35]. Similarly, no studies directly contrasted the use of research evidence before and after the 2013 reorganisation of public health structures, although some did describe the changes in some of the influencers of evidence use, including the changing way in which priorities were set and the influence of local accountability and politicisation [14, 41]. Therefore, with regards to meeting our aims of exploring evidence-use processes and understanding how these processes may have changed as a result of the reforms described earlier, the body of available evidence did not allow us full insight.

Further caveats exist, particularly around the broad inclusion criteria with regards to study design which precluded more formal synthesis and an assessment of individual study quality. However, all but three studies supported multiple themes (Table 3), providing some indication that the majority of the included studies were relevant to the review. The narrow focus on local public health decision-making in England could also mean that the results have limited applicability to other settings; although, the findings have a degree of overlap with those of previous reviews of evidence use in health settings elsewhere [31, 60]. For example, Liverani and colleagues’ [60] conclusion that our understanding of the major influencers on evidence use in public health decision-making remains piecemeal also stand here. Additionally, two studies were identified that took place in Scotland and Wales where the public health challenges may be similar but the policy-making context differed. Nevertheless, both studies provided support for the key themes emanating from this review, and particularly the importance of research evidence that could be reinterpreted to become contextually meaningful, including blending the findings from research evidence with findings from other sources [61, 62].

**Table 3:** Summary of the studies by analytic theme

Study no.	Authors/year	Analytic themes					
		Locality of evidence	Decision-making vs local context	Expert opinion	Evaluation evidence, experts and localism	Economic evidence	Facilitator and barriers
1	Blackman et al. (2011) [59]						●
2	Blackman et al. (2012) [43]	●	●			●	
3	Clarke et al. (2013) [46]	●		●			
4	Hunter et al. (2016) [47]	●	●		●	●	
	Marks et al. (2015) [14]	●	●		●	●	
5	Jenkins et al. (2015) [66]		●				
	Peckham et al. (2015) [44]	●	●				
6	King (2014) [48]				●	●	●
7	Lister and Merritt (2013) [58]					●	●
8	Marsh et al. (2012) [55]				●	●	●
9	Martin et al. (2011) [52]			●			●
10	McGill et al. (2015) [34]	●					●
11	Milton et al. (2014) [56]				●		●
12	Oliver et al. (2012) [51]			●			●
13	Oliver et al. (2013) [50]			●			
14	Oliver and De Vocht (2015) [42]	●		●			●
15	Orton et al. (2011) [53]			●		●	
16	Phillips and Green (2015) [40]	●	●		●	●	
17	Rushmer et al. (2014) [54]			●			
18	Salisbury et al. (2011) [45]	●			●	●	
19	Skinner et al. (2015) [67]	●					
20	Willmott et al. (2015) [49]	●				●	
21	Wye et al. (2015) [41]	●		●	●		●

● = Study provided support to the theme

Consistent across the studies was a tendency to describe research evidence as being underutilised in decision-making but generally to be absent of sufficient detail around the type, process and context of the decision being made that could illuminate a way forward. Many of the suggestions being put forward in studies tend to be from the perspective of researchers, and arguably, this has led to a pre-occupation with identifying different forms of evidence, as opposed to different form and stages of decision-making where evidence is needed but is currently not, or under-, utilised.

### Complexity and heterogeneity in decision-making and public health

Heterogeneity in process and structure is ostensibly a defining characteristic of public health decision-making post-2013. Local decision-makers are keen to emphasise the uniqueness of their areas [40], as opposed to identifying commonalities. It is important that we acknowledge this heterogeneity in processes and structures as part of the ecosystem in which public health services and interventions operate, and in which evidence is used. However, it is also important that we attempt to understand and respond to this heterogeneity. Research has not advanced to understanding the landscape in terms of typologies and groupings of public health decision-making processes and structures. Developing such typologies could facilitate understanding broad patterns of evidence use (and need) within these groupings and ultimately bridge a gap between evidence use and generation. Similarly, the demand for local evidence uncovered [34, 40–42, 62] should be interrogated further.

### A changing balance of factors influencing decision-making

It is difficult to fully establish how the transition into local authorities has influenced the politics of decision-making. Certainly, some studies suggested that political influence had disrupted previously established flows of evidence into different stages of decision-making [24, 40, 48, 49], which may impede on evidence-based policy-making. Some studies suggested that a more politicised environment influenced priority setting [14, 47] and that the local political ideology and the case required for a public health issue changed over a short duration [49]. This may support the idea that more politicised environments could foster cultures of “short-termism” in a priority setting [63] and indicate the way in which a change in administration after an election may lead to rapid changes in priorities; both of which present challenges to evidence production.

The demand for economic evidence, a theme discussed in this review, is likely to strengthen against the background of funding cuts to public health budgets outlined in the introduction. Meanwhile, the role of local experts in a knowledge translation role is also likely to be sustained if research evidence continues to hold either low resonance or transferability or usability among end users. However, there is much left to learn about the role that experts do indeed fulfil, given that one study suggested that experts take a directive role. Regarding our own concern around the role of systematic reviews in public health decision-making, it is notable that almost half of policy actors in one study viewed meta-analyses as useful; although, none reported using this evidence regularly [42].

## Conclusions

The body of available evidence did not allow for full insight on the way in which evidence is used in English public health decision-making; although, a number of distinct processes and preferences were identified. Overall, these findings suggested that much of the research evidence being produced may not match the needs of decision-makers due to its “global” nature and that decision-makers may instead look to other means and sources to bridge this need. A clear gap in the literature identified in this systematic review was an insight into the process of decision-making in the new public health landscape, and how evidence is used differentially at different stages; Phillips and Green [40] provided an exception.

Most of the literature encountered in this review focus on attempting to establish “determinant frameworks” of barriers and facilitators, without first establishing “process models” of the use of evidence [35]. Constructing process models could establish an understanding of who “consumes” research evidence and distinguish between decision-influencers and decision-makers, who may have very different evidence requirements. Some studies, conducted pre-2013, suggested that influential actors in public health decision-making were not necessarily the most senior; and several studies have indicated that external experts also hold an high degree of influence, if not holding decision-making powers themselves. Ascertaining the steps that should be taken to enhance evidence use in public health decision-making is challenging in the absence of detailed understandings of current practice; process models which identify evidence needs at different stages of decision-making for different types of decisions being made could help to overcome this limitation.

The challenges raised in this review are clear. These include the need for researchers to develop a much deeper understanding of evidence requirements from the perspective of decision-makers. The current body of literature and, in particular, the solutions and facilitators to increasing research evidence use identified tend towards an understanding of decision-makers needs from the researchers’ perspective. This means that we prioritise types of evidence that should “fit” into informing decisions, rather than approaching the issue from the perspective of the types of decisions where insights and knowledge from evidence is needed, but where this need is unmet.

## Additional files

Additional file 1: Scoping review on the use of evidence in local authorities. Search strategy and results. (DOCX 17 kb)

Additional file 2: PRISMA 2009 Checklist. (DOC 64 kb)

Additional file 3: Preliminary textual descriptions of studies and their findings. (DOCX 41 kb)

## Abbreviations

CCG: Clinical commissioning group
HSCA: Health and Social Care Act
HWS: Health and Wellbeing Strategy
LA: Local authority
PCT: Primary care trust
