# Supplementary material for: The use of evidence in English local public health decision-making: a systematic scoping review
Source: Implement Sci. 2017 Apr 20;12:53. doi: 10.1186/s13012-017-0577-9 (PMC5399426; doi:10.1186/s13012-017-0577-9)
Supplement: Supplementary file 1 — Scoping review on the use of evidence in local authorities. Search strategy and results. (DOCX 17 kb) [file 13012_2017_577_MOESM1_ESM.docx]

**Scoping review on Use of evidence in Local Authorities. Search Strategy and results (6.6.2016).**

*Scopus = 136*

(TITLE-ABS-KEY ("local authorit*") OR TITLE-ABS-KEY ("local government*") OR TITLE-ABS-KEY ("clinical commissioning") AND TITLE-ABS-KEY ("public health") OR TITLE-ABS-KEY (“Health promotion”) OR TITLE-ABS-KEY ("decision mak*") OR TITLE-ABS-KEY ("policy mak*") OR TITLE-ABS-KEY ("health policy") AND TITLE-ABS-KEY (evidence) AND SUBJAREA (mult OR medi OR nurs OR dent OR heal OR mult OR busi OR deci OR econ OR psyc OR soci) AND PUBYEAR > 2009 AND (LIMIT-TO (AFFILCOUNTRY, "United Kingdom"))

*Pubmed = 394*

(((("Local Government"[Mesh] OR "Public Assistance"[Mesh] OR "Financing, Government"[Mesh]) AND "England"[Mesh]) OR "Great Britain"[Mesh]) AND (("Health Policy"[Mesh] OR "Health Promotion"[Mesh] OR "Health Care Reform"[Mesh]) AND ("Policy Making"[Mesh] OR "Decision Making"[Mesh]))) OR (("local government"[All Fields] AND "public health"[All Fields]) AND ("great britain"[MeSH Terms] OR ("england"[MeSH Terms] OR "england"[All Fields]) OR uk[All Fields])) AND ("2010/08/06"[PDAT] : "2016/06/31"[PDAT])

|  | | **Search Strategy HMIC.6.6.2016** | **Results** | **Type** |
| --- | --- | --- | --- | --- |
|  | | | | |
| 1 | exp local government/ or exp public finance/ | | 4930 | Advanced |
| 2 | exp Health service reform/ or exp health policy/ or exp health promotion/ | | 22244 | Advanced |
| 3 | exp decision making/ or exp policy formulation/ or exp Evidence based policy/ | | 6868 | Advanced |
| 4 | (("local government" or "local author$") and "public health").mp. | | 925 | Advanced |
| 5 | exp england/ or exp Great britain/ | | 24654 | Advanced |
| 6 | 1 or 2 | | 26905 | Advanced |
| 7 | 3 and 6 | | 816 | Advanced |
| 8 | 4 or 7 | | 1727 | Advanced |
| 9 | 5 and 8 | | 158 | Advanced |

| EconLit – 6.06.2016 |
| --- |

| **Search Terms** | **Search Options** | **Actions** |  |
| --- | --- | --- | --- |
| S9 | S5 AND S8 | **Limiters** - Published Date: 20100101-20161231  **Search modes** - Boolean/Phrase | (215) |
| S8 | S4 OR S7 | **Search modes** - Boolean/Phrase | (16,783) |
| S7 | S3 AND S6 | **Search modes** - Boolean/Phrase | (1,861) |
| S6 | S1 OR S2 | **Search modes** - Boolean/Phrase | (49,797) |
| S5 | england or britain or uk or united kingdom | **Search modes** - Boolean/Phrase | (39,940) |
| S4 | "local government" OR "local author$" AND public health | **Search modes** - Boolean/Phrase | (15,502) |
| S3 | decision making OR evidence based policy OR policy making | **Search modes** - Boolean/Phrase | (36,091) |
| S2 | health care reform OR health policy OR health promotion | **Search modes** - Boolean/Phrase | (20,019) |
| S1 | local government OR public finance | **Search modes** - Boolean/Phrase | (30,837) |

*Manual search = 12*

Total number of number of retrieved references = **903**

After removing duplicates = **859**
