# Supplementary material for: The use of evidence in English local public health decision-making: a systematic scoping review
Source: Implement Sci. 2017 Apr 20;12:53. doi: 10.1186/s13012-017-0577-9 (PMC5399426; doi:10.1186/s13012-017-0577-9)
Supplement: Supplementary file 3 — Preliminary textual descriptions of studies and their findings. (DOCX 41 kb) [file 13012_2017_577_MOESM3_ESM.docx]

| Year | Linked study | Study design/ methods | Area | Type of institution | Type of professionals | Before or after 2013 reforms  Key  A=After  B=Before  D=During  N=N/A  U=Unclear | Type of evidence | Target health problem | Summary of the results; Barriers and Facilitators to evidence use |
| --- | --- | --- | --- | --- | --- | --- | --- | --- | --- |
| Martin et al. (2011) |  | Qualitative semi-structured interviews | England/UK | Several: Government (UK), NHS, universities, think tanks, etc. | Commissioners and producers of research relating to public policy and management in the UK | B | Research knowledge in general | General | Study explored the research producer-user divide. Decision-makers agreed that research often fails to fulfil needs, and that greater interaction is required to improve this relationship. Decision-makers offered differing accounts from those of research users around the nature of research knowledge (as a 'product' or a 'mindset'), and of the value, validity and originality in research. The authors argue that there needs to be greater interactions between professionals working in research, policy and practice, but that epistemological barriers did prevent this from happening regularly. |
| Milton et al. (2014) |  | Qualitative interviews | UK | Local Authorities | Collaborators (decision-makers and researchers) involved in joint projects | U | Use and production of evaluation evidence | Housing and health | Examined processes occurring during evaluation of housing interventions. Different ‘cultures of evidence’ were reported but these did not necessarily map onto the public health research/non-academic divide, and did not undermine collaborative work when all parties could gain from taking part in the research. Forming or supporting an existing network of collaborators who can take advantage of windows of opportunity for evaluations, such as was the case in the study where funding was secured for a program of evaluation will facilitate research evidence use and production when accompanied by the commitment, resources and trust to mobilise the larger networks needed to deliver it. |
| K Oliver et al. (2013) |  | Quantitative social network data and qualitative data. | Manchester/ England | Various organisations with a stake in public health | Directors or executives involved in Public Health decision-making | B | Influences and influencers on decision-makers/ research evidence in general | General | Explored the extent to which informal relationships influenced how decision-makers find evidence and make decisions for public health policy (PHP) through collecting social network data. Mid-level managers found to be some of the most influential actors by controlling policy processes through gate keeping key organizations, providing policy content and managing selected experts and executives to lead on policies. Public health professionals and academics were often only indirectly connected to policy via managers. |
| Orton et al. (2011) |  | In-depth qualitative study | Local, regional or national level in the UK | Various organisations with a stake in public health including LAs and voluntary sector | Commissioners, public health professionals, analysts and researchers | B | Influences and influencers on decision-makers/ research evidence in general | General (health inequalities) | Explored influencers in decision-making around health inequalities. The short term target- and outcome-led cultures of decision-making often lead to investment in "downstream" public health interventions, rather than the "upstream" approaches that are most effective at reducing inequalities. Authors identify that researchers have a vital role to play in providing the complex evidence required to compare different models of prevention and service delivery but that one of the main barriers to addressing health inequalities is the medicalisation of the public health system and the over-riding influence of “downstream” targets and outcome |
| Rushmer et al. (2014) |  | A realist evaluation of workshops | UK | Local Authorities, NHS and strategic partnerships | Decision-makers at various levels of seniority | B | Research evidence in general. | A number of defined public health issues | Evaluated the impact of knowledge translation workshops in increasing the use of research evidence in decision-making. The findings suggest that providing evidence summaries, input from academic and practice experts, conversational spaces and personal action planning are necessary to create enthusiasm within workshops, but are insufficient to prompt practice change in the medium term. Barriers to the use of research evidence thought to be systemic, processual and organizational, or concern the capacity of the evidence base to support action-oriented recommendations. Authors tend to suggest that it is the supply of ‘usable’ evidence – evidence that is useful to individuals and evidence that is usable within structures and organisations – that is failing. |
| Salisbury et al. (2011) |  | Critical appraisal and qualitative research around the process of evaluation | England | Local and national NHS commissioners | Local and national NHS commissioners working on different areas including issues around population health (prevention and promotion) | B | Evaluation evidence. | Various health issues (including public health) | Explored the commissioning and intended use of evaluation evidence. Authors identified that there was a commitment to the concept of evaluation but little clarity about how findings would be used. The authors also identify tensions between the priority of evaluators for ensuring methodological rigour and the needs of service providers for swift, contextually relevant findings. Additional concerns were identified about the transparency of methods and results. |
| King (2014) |  | Survey and interviews | England | Local Authorities | Senior local authority officers | B | Economic Evidence | Physical activity | Explores the use of Social Return on Investment (SROI) evidence in LA decision-making around investing in services to increase levels of physical activity. The author identifies that economic (Social Return on Investment) evidence for Local Authorities is most compelling when the business case is established across service areas. SROI evidence is compelling is as: it provides metrics for measuring value of outcomes that are difficult to quantify; it can provide a basis for a pre-emptive approach to operational planning; it can provide a basis for service continuation of those services demonstrating social value; it can stimulate an environment of continuous quality improvement; SROI evidence can provide a basis for stakeholder engagement; enhance accountability; and can help to identify health gains of services focussed on health determinants. |
| KA Oliver and de Vocht (2015) |  | Survey and semi-structured interviews conducted with a sub-sample of the respondents | UK - with concentration around Greater Manchester | Various institutions (survey fielded to various people) | Senior decision-makers, officers, academics and third sector organisations. | A | Research evidence in general. | General | Survey of evidence preferences finds that local data were the most frequently cited type of evidence used and perceived to be useful. Examples of local data ranged from standard surveillance data (morbidity and mortality) to service provision data, to complex models of patient flows and integrated health and social care records. The second most cited source of evidence was the joint strategic needs assessments (which also reflect local data). Other types of local data mentioned were public views, knowledge of local personalities and leadership and tacit knowledge. Research-derived evidence was considered useful and used by most participants, especially qualitative research studies and survey data. Systematic review and trial data were also used (but were much less popular. Two respondents also mentioned evidence summaries or reviews, and Cochrane reviews specifically. Most interviewed actors, however, did not use them and similarly also did not use meta-analyses. The most frequently mentioned sources were governmental websites (84%), followed by National Institute for Health and Care (NICE) guidelines (70%). ‘Experts’ and ‘other people’ were frequently cited as a source of information and evidence although this was not always highly ranked. Some interviewees described ‘personal experience’ as a source of information and evidence. The authors conclude that taken in conjunction with the preference for experts and other people in the categorical answers, it is obvious that interpersonal relations are a major source of knowledge transfer. |
| K Oliver et al. (2012) |  | Documentary analysis and a survey. | Greater Manchester | Various institutions (survey fielded to various people) | Senior decision-makers. | B | Research evidence in general. | General | Explored the interrelationships between decision-makers and the most influential actors. Consistently across the networks explored the best authorities were perceived to be individuals with two main characteristics: Firstly, they had jobs which bridged local authority and NHS organisations. Secondly, they were not chief executives or council leaders but managers involved in public health policy. The authors note that surprisingly, given the rhetorical importance accorded to evidence-based policy in the UK, universities and other information-disseminating bodies were poorly represented. Actors from these types of institutions were rarely connected directly to important or influential actors, nor were they considered influential themselves. The authors recommend that researchers and academics can use network analysis to identify key actors as targets for evidence dissemination. |
| Lister and Merritt (2013) |  | Survey, interviews and the convening of an expert panel | England | Many types of commissioners including Local Authority | Included Local Authority Commissioners among others | B | Economic Evidence | General | Examined the development of tools for local commissioners to base decisions on economic evidence. Authors identify that costs and benefits to all stakeholders including clients, employers, government, and the NHS (beyond commissioner and provider costs) should be taken into account. Emphasis was placed on the need to identify possible savings to Local Authority services in view of changing responsibilities, and a number of obstacles to the production of this evidence were identified. The availability of evidence that was in the metrics of QALYs was not found for all subject areas and was a barrier to the effective use of evidence.  None mentioned - not an evaluation of the tools |
| Clarke et al. (2013) |  | Survey | England | PCTs (which did incorporate Public Health) | All staff employed at NHS grade 7 or above involved in commissioning and decision-making. A third were working in Public Health | B | All types of evidence | Various health issues (including public health) | Survey of decision-making processes and included and examination of the role of evidence. Local public health evidence was identified as being some of the most useful. The most common type of decision reported was ‘changing the organisation or design of a particular service’; this was followed by a ‘major decision on strategic direction’ and Individual Funding Requests (IFRs) |
| Marsh et al. (2013) |  | Documentary analyses and discrete choice experiment | UK | Various involved in public health | Senior decision-makers | B | All types of evidence | A number of defined public health issues | Explored the evidence that decision-makers were interested in obtaining. The results of the review, workshop and survey were combined to identify the following criteria as being important for decision-makers: (i) incremental cost-effectiveness: cost per QALY gained; (ii) the proportion of the population eligible for the intervention; (iii) the distribution of benefits; (iv) affordability: the budget required to fund the intervention if all eligible people received the intervention; (v) certainty: confidence in the evaluation of the intervention. Feasibility and acceptability of interventions was also identified as being important. The authors present an example of how this technique can be used to support decision-making. |
| Phillips and Green (2015) |  | Ethnographic techniques. | Six LAs in England | Six Local Authorities | Various - Officers, Managers, Directors | A | Decision-making involving different types of evidence | Alcohol | Ethnographic study around the culture of decision-making with respect to alcohol. Authors found that local authority officers emphasise their accountability to a number of stakeholders: their local population, new public management and elected councillors. Identified that the culture of decision-making supports the privileging of local knowledge and expertise as a way of understanding problems, designing actions and understanding their value. Officers put local evidence in the form of various data and information into conversation with their experiential expertise to create the knowledge that is used to reassure the public before a decision is made and justify those actions afterwards. Knowledge generated through evaluative activities is embedded in practice and used to develop and adapt their approach in the future, rather than to judge an approach as universally valid or invalid and created a culture of epistemology of practice rather than of evidence. Officers emphasised the unique, rather than the typical, features of their area or population. |
| Willmott et al. (2015) |  | Semi-structured telephone interviews | SE England | Local Authorities | Directors of Public Health (DsPH) | A | All types of evidence | General | Explored how public health decisions were made in new cultures. Even over the short time covered by the study, what DsPH were making the case for shifted and one described how they were initially concerned to: ‘try and get the council to understand what its new public health function was’. The authors identified that DsPH made positive business cases outlining needs, solutions and their effectiveness, cost and economic impact. Interviewees were clear that ‘having the economic argument is hugely important’ and evidence was perceived to be vital in substantiating arguments. The authors described that DsPH felt public health evidence was patchy, especially in demonstrating shorter term impact, savings, effects on wider determinants of health and benefits. The authors also described that interviewees felt that evidence was not always required to support arguments where the case was congruent with current ideas or political ideology. Knowledge translation was also identified as the authors described that DsPH highlighted the importance of making evidence locally relevant, carefully framing their message (in documents or verbally) and of being ‘versed in council terms’ to make ‘things really accessible’ (discussion participant) but at the same time, trying to ‘not come in too expert’ so as not to repel potential allies. |
| Wye et al. (2015) |  | Case study design and ethnographic techniques | England | PCTs, CCGs and LAs | Looked at commissioners of evidence and commercial or not-for-profit providers | D | All types of evidence | General | Explored evidence commissioning practices. Authors identified that local information often trumped generalised research-based knowledge or information from other localities. They also provide examples of where anecdotal evidence had trumped more robust forms of evidence. The authors state that when asked about academic research, participants mainly talked about the difficulties in accessing and using research and describe how one Public Health consultant discussed the difficulties in making research digestible for consumption by commissioners. Although NICE guidance was described as ‘hard edged’ with stronger links to academic research, it was not mandatory to apply, and locally implementation was sometimes problematic if local services did not exist to support the guidelines. |
| Skinner et al. (2013) |  | Qualitative – a series of interviews | Northern focus (but not exclusive) | Various - LAs, NHS etc | Various but included a number of local commissioners | D | Focus particularly on Joint Strategic Needs Assessment (JSNAs) and evidence supporting | Focus on Black and Minority Ethnic health | Issues identified with the specificity and relevance of JSNAs for BME people; identified broader issues of obtaining data to support commissioning for BME and other minority populations |
| Blackman et al. (2011) |  | Survey | England | PCTs and LAs | Completed by public health professionals, clinicians and local authority health leads | B | All types of evidence | Mortality inequality in terms of cancer and CVD | The results here attempt to examine the decision-making processes associated with highly successful implementation and those associated with less successful implementation. The study finds that greater levels of 'process' in decision-making can lead to lower levels of narrowing of the gap - which does include some elements of developing 'evidence-based strategies'. |
| Jenkins et al. (2015) | Peckham et al. (2015) | An online survey | England | LAs | Directors of Public Health | A | All types - influencers around decision-making; all types of evidence | Various | Survey examines DPH as influencers in decision-making and also emphasises HWBs as important documents in influencing decisions. Most DPH (66%) felt ‘quite often able’ to influence priorities in their authority. Almost half felt more able to influence the work of others such as local workplaces or schools. Respondents’ abilities to influence local authority’s priorities were also associated with a requirement by other departments to collaborate with public health on their plans, with HWBs being clearly instrumental in identifying health priorities, and the council’s cabinet engaging in the process of approving public health business plans. |
| Marks et al. (2015) | Hunter et al. (2016) | Workshops,  semi-structured interviews | England | HWBs and stakeholders associated with | Various tiers of decision-maker | A | All types - influencers around decision-making; all types of evidence | Various | Four influences on prioritizing public health investment are described: (i) organizational context; (ii) commissioning and priority-setting context; (iii) views of evidence; and (iv) understandings of public health. Organisational context includes accountability to the local electorate as well as poorly defined visions of what 'success' looks like. The authors identify that decision-support methods need to be considered in the context of local government priority-setting and commissioning processes and current options could be viewed as bureaucratic including; also broader concerns about austerity are discussed. There is ambiguity as to what counts as evidence - perceptions of local people is viewed or at least treated as evidence of need even if it's quite removed from the messages from other sources. |
| Blackman et al. (2012) |  | Semi-structured interviews | England, Wales and Scotland (UK) | PCTs, the local authorities and local strategic partnerships (LSPs). | Senior professionals | B | General research evidence | Health inequalities | The authors find three different national policy regimes create contrasting contexts, especially regarding the different degrees of emphasis in these regimes on audit and performance management. They find that politics dominates how health inequalities are framed for intervention, affecting their prioritisation in practice and how audit, evidence and treatment are described as deployed in local strategies. |
| Hunter et al. (2016) | Marks et al. (2015) | Semi-structured interviews | England | Local Authorities | Various senior decision-makers | A | General research evidence | To explore the use of prioritisation tool in Public Health spending decision | The authors explored prioritisation tools and found many respondents expressed an interest in prioritisation tools although some scepticism was expressed about their value and impact on decision-making. The authors’ findings suggest that the adoption of priority-setting tools in decision-making processes in public health poses some significant challenges due to the influence of contextual factors within local government and identify certain enabling factors that tend to be present. |
| McGill et al. (2015) |  | Qualitative – focus groups | England: London and North West; International | Local government officers (NB: designated as planning etc but may be working on public health projects) | Manager to director level (n=15 in total) | A | General research evidence | Social determinants of health - interest was on leisure, planning, construction and relationships with health | The authors identify a number of predominant themes around the use of research evidence. Theme 1 included multiple conceptualisations of evidence and participants across the three workshops held a narrow view of health research as ‘pure science’ (ie, driven by high quality empirical research) compared with the built environment as a form of creative ‘art’, a philosophy or in one participant’s words, a ‘theology’. Where evidence could move beyond textual - e.g. drawings etc. They found participants from all three focus groups gave examples of quantitative and qualitative data used to inform their decisions, but again the kind of evidence they referred to was not simply analogous to academic research. Theme 2 revolved around the distinction of ‘viability’ versus outcomes: Participants frequently spoke about the need to demonstrate the ‘viability’ of interventions and services, with the focus on whether initiatives could be delivered, sustained and accepted by stakeholders and users. Theme 3 supported other studies and was based on locally relevant evidence: Participants placed knowledge about their local area at a premium. |
| Peckham et al. (2015) | Jenkins et al. (2015) | Surveys and interviews to support case study | England | LAs | DPH, PH consultants, elected members, chief executives | A | General research evidence | Various | Study examining public health decision-making in new structures. Found most DsPH reported that the ring-fenced public health budget had been used to invest in other local authority departments, whereas only 65% of elected members thought this was the case. The authors described that in influencing the system, participants often talked about using specific relationships as levers. While there was an awareness of political accountability, this differed according to role and councillors were said to view the public from a particular perspective that is informed by experience and contact. This could mean that the way in which public health issues were prioritised were often more informal compared to the more formal needs assessments used by public health staff. Respondents in the study claimed that elected members were felt to bring more granular knowledge and could provide a bit of a ‘sense-checker’. |

Blackman, T., Harrington, B., Elliott, E., Greene, A., Hunter, D.J., Marks, L., et al. (2012). Framing health inequalities for local intervention: comparative case studies. *Sociology of health & illness,* 34, 49-63.

Blackman, T., Wistow, J., & Byrne, D. (2011). A qualitative comparative analysis of factors associated with trends in narrowing health inequalities in England. *Social science & medicine,* 72, 1965-1974.

Clarke, A., Taylor-Phillips, S., Swan, J., Gkeredakis, E., Mills, P., Powell, J., et al. (2013). Evidence-based commissioning in the English NHS: who uses which sources of evidence? A survey 2010/2011. *BMJ open,* 3.

Hunter, D., Marks, L., Brown, J., Scalabrini, S., Salway, S., Vale, L., et al. (2016). The potential value of priority-setting methods in public health investment decisions: qualitative findings from three English local authorities. *Critical Public Health*, 1-10.

Jenkins, L.M., Bramwell, D., Coleman, A., Gadsby, E.W., Peckham, S., Perkins, N., et al. (2015). Integration, influence and change in public health: findings from a survey of Directors of Public Health in England. *Journal of Public Health*, fdv139.

Katikireddi, S.V., Bond, L., & Hilton, S. (2014). Perspectives on econometric modelling to inform policy: a UK qualitative case study of minimum unit pricing of alcohol. *Eur J Public Health,* 24, 490-495.

King, N. (2014). Making the case for sport and recreation services: The utility of social return on investment (SROI) analysis. *International Journal of Public Sector Management,* 27, 152-164.

Lister, G., & Merritt, R. (2013). Evaluating the value for money of interventions to support behavior change for better health (behavior change evaluation tools). *Social Marketing Quarterly,* 19, 76-83.

Marks, L., Hunter, D., Scalabrini, S., Gray, J., McCafferty, S., Payne, N., et al. (2015). The return of public health to local government in England: changing the parameters of the public health prioritization debate? *Public health,* 129, 1194-1203.

Marsh, K., Dolan, P., Kempster, J., & Lugon, M. (2013). Prioritizing investments in public health: a multi-criteria decision analysis. *J Public Health (Oxf),* 35, 460-466.

Martin, G., Currie, G., & Lockett, A. (2011). Prospects for knowledge exchange in health policy and management: institutional and epistemic boundaries. *J Health Serv Res Policy,* 16, 211-217.

McGill, E., Egan, M., Petticrew, M., Mountford, L., Milton, S., Whitehead, M., et al. (2015). Trading quality for relevance: Non-health decision-makers' use of evidence on the social determinants of health. *BMJ open,* 5.

Milton, S., Petticrew, M., & Green, J. (2014). Why do local authorities undertake controlled evaluations of health impact? A qualitative case study of interventions in housing. *Public health,* 128, 1112-1117.

Oliver, K., & de Vocht, F. (2015). Defining 'evidence' in public health: a survey of policymakers' uses and preferences. *Eur J Public Health*.

Oliver, K., De Vocht, F., Money, A., & Everett, M. (2013). Who runs public health? A mixed-methods study combining qualitative and network analyses. *Journal of Public Health (United Kingdom),* 35, 453-459.

Oliver, K., Everett, M., Verma, A., & de Vocht, F. (2012). The human factor: Re-organisations in public health policy. *Health Policy,* 106, 97-103.

Orton, L.C., Lloyd-Williams, F., Taylor-Robinson, D.C., Moonan, M., O'Flaherty, M., & Capewell, S. (2011). Prioritising public health: A qualitative study of decision making to reduce health inequalities. *BMC public health,* 11.

Peckham, S., Gadsby, E., Coleman, A., Segar, J., Perkins, N., Jenkins, L., et al. (2015). PHOENIX: Public Health and Obesity in England–the New Infrastructure examined. London: London School of Hygiene and Tropical Medicine.

Phillips, G., & Green, J. (2015). Working for the public health: Politics, localism and epistemologies of practice. *Sociology of Health and Illness,* 37, 491-505.

Rushmer, R.K., Hunter, D.J., & Steven, A. (2014). Using interactive workshops to prompt knowledge exchange: A realist evaluation of a knowledge to action initiative. *Public health,* 128, 552-560.

Salisbury, C., Stewart, K., Purdy, S., Thorp, H., Cameron, A., Lart, R., et al. (2011). Making the most of evaluation: a mixed methods study in the English NHS. *J Health Serv Res Policy,* 16, 218-225.

Skinner, J., Salway, S., Turner, D., Carter, L., Mir, G., Bostan, B., et al. (2013). Aligning JSNA and EDS: Benefits for minority ethnic communities? *Journal of Integrated Care,* 21, 77-90.

Willmott, M., Womack, J., Hollingworth, W., & Campbell, R. (2015). Making the case for investment in public health: experiences of Directors of Public Health in English local government. *J Public Health (Oxf)*.

Wye, L., Brangan, E., Cameron, A., Gabbay, J., Klein, J., & Pope, C. (2015). Knowledge exchange in health-care commissioning. *Knowledge exchange in health-care commissioning: case studies of the use of commercial, not-for-profit and public sector agencies, 2011-14*. Southampton (UK): NIHR Journals Library
